# Forecasting psychomotor vigilance test performance from facial videos

**DOI:** 10.1093/sleep/zsaf220

**Published:** 2025-07-30

**Authors:** Takashi Abe

**Affiliations:** International Institute for Integrative Sleep Medicine (WPI-IIIS), Tsukuba Institute for Advanced Research (TIAR), University of Tsukuba, Tsukuba, Japan

The psychomotor vigilance test (PVT) is highly sensitive to sleep loss and to the restorative effects of napping^1–4^ and has therefore been a widely used tool for assessing alertness in sleep science and sleep medicine, not only in laboratory studies but also in real-world settings, including space missions.^5–7^ The PVT assesses behavioral alertness by measuring reaction times to a digital counter appearing on a screen at unpredictable intervals of 2–10 s. Participants press a response button as quickly as possible upon seeing the stimulus. A typical session lasts 10 min.^8^ Accurately predicting when performance on the PVT will decline enables intervention before attention lapses occur. Interventions, such as planned sleep opportunities, brief naps, or targeted caffeine use, can then be scheduled proactively rather than reactively. Therefore, the development of mathematical models capable of forecasting PVT performance has become a major focus of fatigue management research.

Previous attempts have used a seminal two-process model of sleep–wake regulation^9^ to predict bout-to-bout fluctuations in PVT performance. However, this model often fails for some participants because the PVT responses to sleep loss differ markedly between individuals.^10^^,^^11^ Therefore, starting from the pioneering work of Van Dongen et al.,^12^ biomathematical models have been developed to forecast an individual’s PVT performance based on their own previous PVT results. Notably, Reifman’s group extensively developed and validated the unified model of performance (UMP). This model forecasts an individual’s PVT outcomes not only during total sleep deprivation, but also under chronic sleep restriction and after caffeine ingestion, by integrating the individual’s past PVT results with their sleep and caffeine histories.^13^^,^^14^ A recent algorithm based on the UMP has successfully optimized sleep schedule and caffeine consumption to maximize alertness.^15^ A smartphone application, “2B-Alert App 2.0,” has also been developed, which provides personalized caffeine schedules based on each individual’s PVT profile. This demonstrates that PVT forecasting has reached a stage where it can be implemented in real-world settings.^16^

Despite their potential, PVT forecasting models still face some challenges in real-world applications. One of the major challenges is that administering PVT every few hours requires the interruption of the user’s ongoing activities, such as driving. Although shortened versions of the PVT, such as the adaptive 3-min PVT,^17^ are available, testing still requires users to briefly pause their current activities. Thus, in real-world settings, the administration of PVT multiple times per day for forecasting purposes may not be feasible.

In this issue, Subramaniyan et al.^18^ present an innovative study titled “Personalized alertness prediction using video-based ocular and facial features.” This study introduces a novel approach to personalized PVT forecasting that does not require the administration of the PVT itself. Instead of using actual PVT measurements, this method uses an estimated PVT mean reaction time derived from facial videos as input to the UMP, which in turn predicts the individual’s subsequent performance. The authors evaluated the approach in a 62-h total sleep deprivation study involving 26 participants, during which a 5-min PVT was administered every 3 h. Data from the first 42 h were analyzed. After each PVT, a 3-min facial video was recorded, and the first minute of each video was used to estimate the mean PVT reaction time from the preceding test. A linear mixed-effects model was used to estimate the mean PVT reaction time as a continuous variable using ocular and facial features extracted from the video. Of the 15 ocular and facial features analyzed, PERCLOS (percent time of slow eyelid closures) was the most informative indicator for estimating PVT performance, confirming earlier findings that PERCLOS is the strongest physiological correlate of PVT outcomes.^19^ Following PERCLOS, the baseline eye-opening level and then the eyelid-closing velocity were identified as the next most important indicators for estimating PVT mean reaction time. They showed that using these facial-feature-based PVT estimates as UMP inputs resulted in a lower prediction error (root-mean-square error) than when using group-averaged PVT data, although the error remained higher than when actual PVT measurements were used. Thus, providing the UMP with estimated PVT values yields greater predictive benefit than using group-level PVT performance data. Importantly, when the model used the estimated PVT as the input, its prediction error was comparable to the within-subject variability observed in well-rested participants. This result indicates that the model is unlikely to misclassify a severely low arousal level as a normal state of alertness.

To the best of my knowledge, this is the first study to forecast PVT performance using biometric-derived PVT estimates as predictors. In recent years, evidence has accumulated that PVT performance is associated with driving performance,^20^ performance on other tasks such as the Go/No-Go task,^21^ and composite measures from subjective scales, simple addition calculations, and the Digit Symbol Substitution Test.^22^ Furthermore, among physicians, burnout and depression show a stronger association with PVT performance than with sleep duration.^23^ This approach not only predicts alertness levels but also provides insight into relevant aspects of cognitive and psychological functioning. The approach proposed in this study could become a key component of fatigue management strategies.

However, several issues should be addressed to enhance the effectiveness of this approach in real-world applications. First, this study relied on data collected under conditions of total sleep deprivation in a controlled laboratory setting. Therefore, it is essential to demonstrate the applicability of this technique in diverse, real-world contexts through prospective interventional studies. Recent studies involving real-world shift workers have shown that providing personalized sleep and nap recommendations, derived from each worker’s sleep history, desired sleep onset, and work schedule, can decrease subjective sleepiness in those who closely follow the recommendations.^24^^,^^25^ However, the study targeted subjective sleepiness, which can diverge from objective behavioral performance.^26^^,^^27^ Therefore, real-world prospective interventions aimed at improving PVT performance are an important next step. Second, as demonstrated by Subramaniyan et al.,^18^ the accuracy of estimating PVT performance from facial video recordings remains relatively low. Despite its limited accuracy, adding the estimated PVT to the UMP allowed it to forecast PVT performance. In recent years, machine learning models for estimating PVT performance have attracted increasing attention. These models use physiological metrics such as eye and eyelid movements,^28^ heart rate,^29^ or electroencephalographic signals.^30^ These techniques for inferring PVT performance from physiological markers still require further improvements in accuracy and additional validation under real-world conditions. Nevertheless, once these methods advance sufficiently, integrating them with UMP could provide even more precise forecasts of PVT performance.

In conclusion, the model developed by Subramaniyan et al.^18^ demonstrated the capability of forecasting PVT performance by combining passive facial recordings with sleep history data, offering a novel proof-of-principle. Accurately predicting PVT performance in advance without administering the test could be a valuable tool for counteracting real-world declines in alertness and, in turn, averting related accidents and performance deficits across diverse domains where maintaining optimal alertness is paramount.

## Disclosure statement

*Financial disclosure*: The author has no financial disclosures.

*Non-financial disclosure*: The author has no non-financial disclosures.
